# Chinese Herbal Medicine for Treating Epilepsy

**DOI:** 10.3389/fnins.2021.682821

**Published:** 2021-07-02

**Authors:** Chia-Hui Lin, Ching-Liang Hsieh

**Affiliations:** ^1^Department of Chinese Medicine, China Medical University Hospital, Taichung, Taiwan; ^2^Chinese Medicine Research Center, China Medical University, Taichung, Taiwan; ^3^Graduate Institute of Acupuncture Science, College of Chinese Medicine, China Medical University, Taichung, Taiwan

**Keywords:** Chinese herbal medicine, epileptic seizure, antiepileptic effect, complementary therapy, diet therapy

## Abstract

Chinese herbal medicine has a long history of use for treating epilepsy. Because of the side effects of Western antiepileptic therapy and the quest for more accessible treatment, complementary and alternative medicines have become popular. Traditional Chinese medical diet therapy appears to be safe and effective. We searched PubMed and the Cochrane Library through November 2020 for the use of traditional Chinese medicine in clinical settings, including plants, fungi, and animals. Combinations of keywords included “epilepsy,” “seizure,” “antiepileptic,” “anticonvulsive,” “Chinese herbal medicine,” “Chinese herb,” and each of the Latin names, English names, and scientific names of herbs. We also summarized the sources and functions of these herbs in Chinese medicine. Different herbs can be combined to increase antiepileptic effects through various mechanisms, including anti-inflammation, antioxidation, GABAergic effect enhancement, modulation of NMDA channels and sodium channel, and neuroprotection. Despite reports of their anticonvulsive effects, adequate experimental evidence and randomized controlled clinical trials are required to confirm their antiepileptic effects.

## Introduction

Epilepsy is a common and chronic neurological disease. The etiologies of epilepsy are defined as structural, genetic, infectious, metabolic, immune, and unknown, which are proposed from International League Against Epilepsy classification system in 2017 (Scheffer et al., 2017). The incidence and prevalence of epilepsy are higher in low- and middle-income countries than in high-income countries, with approximately 80% of patients with epilepsy living in low- and middle-income countries (Meyer et al., 2010; Beghi, 2020). The disease burden could be reduced by improving access to effective treatment (Beghi, 2020).

The pathogenesis of epilepsy is that abnormal electrical discharges derived from the brain including hippocampal, neocortical, cortico-thalamic and basal ganglia networks (Moshé et al., 2015). Though the causes of epilepsy are not totally clear, some possible mechanisms of epilepsy are proposed in many studies. Neurotransmitters, synapses, receptors, ion channels, inflammatory cytokines, immune systems, glial cells, oxidative stress, apoptosis, mitochondrial dysfunction, gene mutations, glycogen and glucocorticoids metabolisms are involved in the pathogenesis of epilepsy (He et al., 2021). Gamma amino butyric acid (GABA) is inhibitory neurotransmitter, and glutamate is excitatory one. Among three types of GABA receptors, GABA_A_ receptors control chloride ion influx, and GABA_B_ receptors increase potassium outflow currents and reduce calcium entry. The activation of GABA receptors makes the inhibitory effect on neuronal membrane potential. Glutamate acts on alpha-amino-3-hydroxy-5-methyl-4-isoxazole-propionate (AMPA) receptors, kainite receptors, and *N*-methyl-D-aspartate (NMDA) receptors. The increased activity of NMDA receptors makes Ca^2+^ influx. Seizures and neuronal damages may occur when the imbalance of inhibitory and excitatory neural activity. Nicotine acetyl cholinergic (nACh) receptors and 5-Hydroxytryptamine (5-HT) receptor also control neuronal excitability and involve in epilepsy (Iha et al., 2017; Zhao et al., 2018). SCN1A, SCN2A, SCN3A, and SCN8A genes which individually encode voltage-gated sodium channels, that is Na_V_1.1, Na_V_1.2, Na_V_1.3, and Na_V_1.6, are related to early onset epilepsies (Brunklaus et al., 2020). Other mutations in ion channels, such as KCNMA1, KCNQ2, KCNT1, KCNQ3, CACNA1A, CLCN2, and HCN1-4, affect the transportation of potassium, calcium, chloride, and cyclic nucleotide (He et al., 2021). Inflammation is the cause and the consequence of seizure, becoming a vicious circle and leading epilepsy to develop and deteriorate (Vezzani et al., 2011). Both infectious and non-infectious inflammatory responses shared common immune pathways then contribute to epilepsy (Vezzani et al., 2016). Oxidative stress and mitochondrial dysfunction could also be the causes and the results of genetic and acquired epilepsies by damaging proteins, lipids, DNA, enzymes, and changing the neuronal excitability (Pearson-Smith and Patel, 2017). Oxidative stress and mitochondrial dysfunction induce apoptosis then led to neuronal death (Méndez-Armenta et al., 2014).

Epilepsy therapies contain anti-epilepsy medication, resective surgery and functional surgery, and medication is the major therapy. Currently approved anti-epilepsy drugs mainly target voltage-gated ion channels such as sodium, potassium, and calcium channels, to modulate the electrical firing of the neuron. For examples of this kind of drugs are phenytoin, carbamazepine, valproate, retigabine, ethosuximide, zonisamide, and so on. Some drugs such as benzodiazepines, barbiturates, and tiagabine act on GABA transporters and GABA receptors to enhance the synaptic inhibition. Vigabatrin inhibit the GABA transaminase to reduce the metabolism of GABA. Some drugs act on ionotropic glutamate receptors, such as perampanel and topiramate act on AMPA glutamate receptors or kainate receptors, and felbamate inhibit NMDA receptors, to suppress the synaptic excitation. Levetiracetam and brivaracetam bind to synaptic vesicle glycoprotein 2A (SV2A) to inhibit the release of glutamate (Wang and Chen, 2019).

Numerous herbal medicines, such as *Ginkgo biloba* and *Huperzia serrata*, have been reported to have antiepileptic or proconvulsant effects (Saxena and Nadkarni, 2011; Sahranavard et al., 2014; Ekstein, 2015; Kakooza-Mwesige, 2015; Shaikh, 2015; Xiao et al., 2015; Cai, 2017; Wei et al., 2017; Manchishi, 2018). The first anti-epilepsy medication resourced from plant is cannabidiol, which is approved by the United States Food and Drug Administration in 2018 for treating Dravet syndrome and Lennox-Gastaut syndrome (Samanta, 2019). Cannabidiol is a non-psychoactive agent of cannabis that is widely studied and proved for its efficacy and safety. Whether the mechanisms of its antiepileptic effect are not fully known, large amounts of clinical trials revealed its potential of medical use (Silvestro et al., 2019). But this new anti-epilepsy drug is expensive and less accessible in most countries because cannabis legalization and medical cannabis are still controversial issues.

Anti-epilepsy drugs have some adverse effects on patients’ quality of life. A latest review article generalized four challenges of anti-epilepsy drugs, including general side effects, psychological challenges, social challenges, and economic challenges (Mutanana et al., 2020). The adverse effects of antiepileptic drugs include severe psychiatric, cognitive, behavioral, endocrine, and dermatological diseases and dysfunctions (Ekstein, 2015; Cai, 2017; Chen B. et al., 2017). The medications may affect the performance of patients’ schoolwork, task, work, and may impede their marriage, and interpersonal relationship. Depression and suicidal ideation are related to increase the dose of anti-epilepsy drugs (Wen et al., 2010). For people who needs long-term treatment of epilepsy, some of them give up the unaffordable and inaccessible anti-epilepsy drugs. Those challenges make patients escape from the treatment with Western medicine, especially seen in the developing countries. Otherwise, even that there are many novel anti-epilepsy drugs developed in recent 20 years, about one-third of patients are lacking appropriate seizure control due to pharmacoresistance (Wang and Chen, 2019). Currently, preventing epileptogenesis and treating comorbidities of epilepsy other than purely symptomatic control of seizures are the remaining challenges (Kobow et al., 2012; Terrone et al., 2016).

Natural medicine has found less side-effects and good efficacy in treating epilepsy. The mechanisms of natural medicine have been reported, including the regulation of synapses, receptors, and ion channels, the inhibition of inflammation, and the regulation of immune system. Natural medicine also can correct the glial cells, improve mitochondrial dysfunction and oxidative stress, and regulate apoptosis (He et al., 2021). Chinese herbal medicine (CHM) has become a popular complementary and alternative medicine. The trend of seeking traditional Chinese medicine for treatment is caused by patients’ fear of the side effects of surgery or Western medication (Ekstein, 2015; Kakooza-Mwesige, 2015). Traditional herbal medicine is also cheaper than mainstream therapy and could be more accessible to patients.

Chinese herbal medicine has been used to treat seizures and epilepsy for thousands of years. Traditional Chinese medicine is based on the theory that medicine and food come from the same sources. Therefore, people can consume herbal medicine in their daily diet. This practice is known as medical diet therapy. Medical diet therapy is the concept of combining nutrition and medicine to treat disease through eating (Wu and Liang, 2018).

The effectiveness of CHMs has also been demonstrated in recent studies. CHM is personalized medicine prescribed based on the constitution theory of Chinese medicine to maintain health and treat diseases (Li et al., 2019). Therefore, individuals may receive different herbal therapies for the same diagnosis.

The aim of this review is to summarize the clinical use and mechanisms of antiepileptic CHM and provide evidence for the efficacy of medical diet therapy, which warrants further exploration.

## Materials and Methods

**FIGURE 1:**
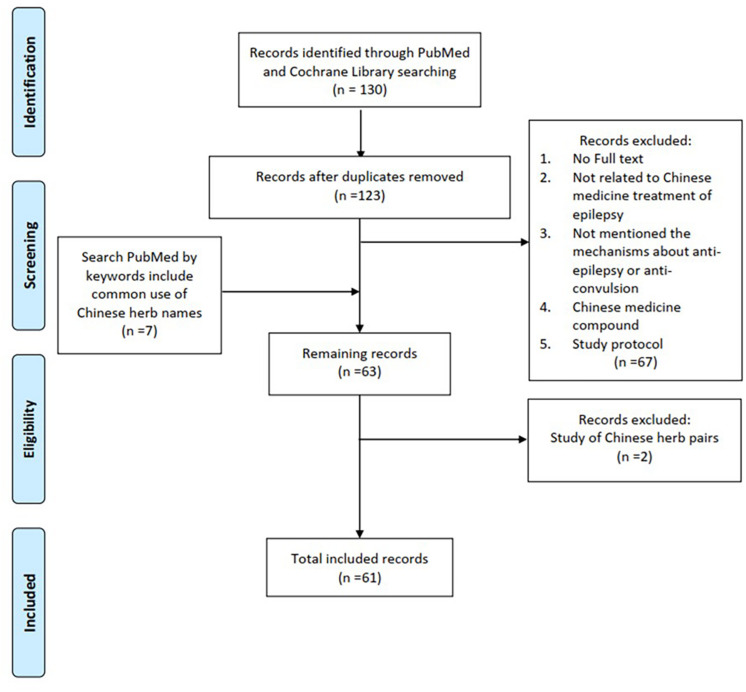
Flow diagram of the literature search process.

Common clinical used CHMs for treating epilepsy and seizure were searched and reviewed in PubMed and Cochrane Library. The various combinations of keywords included the terms “epilepsy,” “seizure,” “antiepileptic,” “anticonvulsive,” “Chinese herbal medicine,” “Chinese herb,” and each of the Latin names, English names, and scientific names of herbs. The search process is presented in Figure 1. The sources of these antiepileptic herbs are summarized in Table 1 based on Taiwan’s official herbal pharmacopeia, third edition (Taiwan Herbal Pharmacopeia 3rd Edition Committee, 2019).

**TABLE 1:** Related functions and possible mechanisms of antiepileptic herbs.

Herbs	Sources	Components	Functions	Possible mechanisms
*Gastrodia elata*	Dried tuber	Vanillyl alcohol Gastrodin Parishin *Gastrodia elata* extract 4-Hydroxybenzyl alcohol 4-Hydroxybenzaldehyde and analogs	Anticonvulsion Anti-inflammation Antioxidation Neuroprotection	(1) Regulate AP-1 expression through the JNK signaling pathway (2) Prevent NMDA excitotoxicity (3) Balance the activity of GABA and glutamate (4) Modulate the MAPK-associated inflammatory responses (5) Inhibit Nav1.6 sodium currents
*Uncaria rhynchophylla*	Dried stem with hook	Rhynchophylline	Anticonvulsion Antioxidation Neuroprotection	(1) Modulate the expressions of MIF and cyclophilin A (2) Reduce the expression of JNKp of MAPK signal pathways (3) Inhibit Nav1.6 I_NaP_ and NMDA receptor currents (4) Reduce GFAP, S100B proteins, and RAGE (5) Attenuate mossy fiber sprouting and astrocyte proliferation (6) Prevent neuron death (7) Regulate TLR and neurotrophin signaling pathways (8) Inhibit the expression of IL-1 and BDNF genes
*Acori tatarinowii*	Dried rhizome	Volatile oil α-asarone β-asarone Eudesmin	Anticonvulsion Antiapoptosis Neuroprotection	(1) Protect GABA-immunoreactive neurons (2) Modulate GABA_A_ receptors then enhance tonic GABAergic inhibition (3) Increase GABA, and reduce glutamate (4) Increase neurotrophic factors by triggering the PKA signaling pathway (5) Modulate Caspase-3 and Bcl-2
*Paeonia lactiflora*	Peeled and dried root	Paeoniflorin	Anticonvulsion Neuroprotection	(1) Suppress the elevation of c-Fos protein, and increase transthyretin and phosphoglycerate mutase 1 (2) Suppress the elevation of glutamate-induced intracellular Ca^2+^
*Bupleurum chinense*	Dried root	Saikosaponin a	Anticonvulsion Neuroprotection	(1) Inhibit NMDA receptor current, I_NaP_ (2) Suppress mTOR signaling pathway (3) Increase Kv4.2-mediated IA
*Ziziphus jujuba*	Dried ripe seed	Hydroalcoholic extract	Anticonvulsion Antioxidation	Increase AChE and BChE activity
*Pinellia ternata*	Dried tuber	Pinellia total alkaloids	Anticonvulsion	(1) Modulate GABAergic system (2) Upregulate GABA_A_ receptors
*Paeonia suffruticosa*	Dried bark of root	Paeonol	Anticonvulsion Antiapoptosis Antioxidation Neuroprotection	(1) Inhibit the expression of proapoptosis factor cleaved caspase-3 (2) Reduce oxidative stress
*Stephania tetrandra*	Dried root	Tetrandrine	Anticonvulsion Antiapoptosis Neuroprotection	(1) Block voltage-gated Ca^2+^ channels (2) Increase the expression of Bcl-2, and reduce the expression of Bax (3) Reduce the expression of multidrug-resistant protein *P*-gp
*Cistanche deserticola*	Dried fleshy stem with scale leaves	Echinacoside	Anticonvulsion Antiapoptosis	(1) Inhibit glutamate excitotoxicity and autophagy (2) Suppress inflammation (3) Activate the Akt/GSK3β signaling (4) Reduce spontaneous glutamate release
*Corydalis yanhusuo*	Dried tuber	DL-Tetrahydropalmatine	Anticonvulsion	Inhibit amygdaloid dopamine release
*Salvia miltiorrhiza*	Dried root and rhizome	Salvianolic acid B	Anticonvulsion Antiapoptosis Neuroprotection	(1) Activate the Akt/CREB/BDNF signaling pathways (2) Upregulate GDNF expression
*Ganoderma lucidum*	Fruiting body	Polysaccharides Ganoderic acid	Anticonvulsion Antiapoptosis Neuroprotection	(1) Stimulate CaMK II α expression to reduce neuronal excitability (2) Inhibit the expression of *N*-cadherin (3) Promote NT-4 expression (4) Enhance the expression of BDNF and TRPC3 (5) Inhibit mossy fiber sprouting (6) Aid neurons plasticity and synaptic reconstruction
*Buthus martensii*	Dried body	Antiepilepsy peptide	Anticonvulsion	(1) Interact with SNAP-25 and NMDA (2) Inhibit Nav1.6 currents to suppress neuronal excitability
*Bombyx mori*	Dried body of the larva	Beauvericin Ammonium oxalate Protein-rich extracts Other ethanol extracts	Anticonvulsion Antiapoptosis Antioxidation Neuroprotection	(1) Regulate the PI3K/Akt signaling pathways (2) Reduce IL-1β, IL-4, and TNF-α, and increase 5-HT and GABA (3) Reduce intracellular Ca^2+^ levels to prevent neuronal signaling
*Cryptotympana atrata*	Dried exuviae of nymph	Water extracts Ethanol extracts	Anticonvulsion	(1) Inhibit GABA in the brainstem (2) Inhibit glycine in the spinal cord

*AP-1, activator protein 1; JNK, c-Jun N-terminal kinases; NMDA, *N*-methyl-D-aspartate; GABA, gamma-aminobutyric acid; MAPK, mitogen-activated protein kinase; MIF, migration inhibitory factor; JNKp, c-Jun aminoterminal kinase phosphorylation; I_NaP_, persistent sodium currents; GFAP, glial fibrillary acidic protein; RAGE, receptor for advanced glycation end products; TLR, toll-like receptor; IL, interleukin; BDNF, brain-derived neurotrophin factor; PKA, cAMP-dependent protein kinase; mTOR, mammalian target of rapamycin; Kv4.2-mediated IA, Kv4.2-mediated A-type voltage-gated potassium currents; AChE, acetylcholinesterase; BChE, butyrylcholinesterase; *P*-gp, *P*-glycoprotein; Akt, protein kinase B; GSK, glycogen synthase kinase; CREB, cAMP response element binding protein; GDNF, glial derived neurotrophic factor; CaMK II α, Ca^2+^/calmodulin-dependent protein kinase II α; NT, neurotrophin; TRPC, transient receptor potential canonical; SNAP, synaptosomal-associated protein; PI3K, phosphoinositide 3-kinases; TNF, tumor necrosis factor; 5-HT, 5-hydroxytryptamine.*

## Results and Discussion

### Plants

#### Gastrodia elata

*Gastrodia elata* is a widely used traditional Chinese medicine for treating neurological disorders, such as headache, insomnia, and epilepsy (Zhan et al., 2016; Liu et al., 2018). *G. elata* has anticonvulsive, anti-inflammatory, neuroprotective, antiapoptosis, and antioxidative effects (Hsieh et al., 2001; Zhan et al., 2016; Liu et al., 2018). In a rat model of ferric-chloride-induced epileptic seizure, Vanillyl alcohol, a component of *G. elata*, suppressed seizures and lipid peroxidation. The pretreatment with either 200 mg/kg or 100 mg/kg Vanillyl alcohol significantly reduced the number of wet dog shakes. The Vanillyl alcohol 200 mg/kg group had significantly greater suppress effect on lipid peroxidation than the Vanillyl alcohol 100 mg/kg group and phenytoin 10 mg/kg group (Hsieh et al., 2000). In a rat model of kainic acid-induced epilepsy, *G. elata* can suppress epileptic attacks by regulating the c-Jun N-terminal kinases (JNK) signal pathway and activator protein 1 (AP-1) expression. Both pre-treatment and post-treatment with *G. elata* modulated phosphorylated JNK and c-Jun protein. However, comparing pre-treatment and post-treatment with *G. elata*, only pretreatment with *G. elata* changed the levels of c-Fos protein, JNK protein, phosphorylated extracellular signal-regulated kinase, and p38 proteins (Hsieh et al., 2007).

One component of *G. elata*, gastrodin, did not act on ionotropic glutamate receptors to inhibit *N*-methyl-D-aspartate (NMDA) receptor–facilitated seizures but did achieve neuroprotective effects through preventing NMDA excitotoxicity that is evaluated on rat hippocampal slice (Wong et al., 2016). Liu et al. reviewed the effects of Gastrodin, and summarized the mechanisms of Gastrodin including the modulation of neurotransmitters, antioxidative, anti-inflammatory, inhibition of microglial activation, regulating mitochondrial function, and up-regulating neurotrophins. Gastrodin has the ability to balance the activity of gamma-aminobutyric acid and glutamate (Liu et al., 2018). Gastrodin also modulated mitogen-activated protein kinase (MAPK)-associated inflammatory responses and inhibited Na_v_1.6 sodium currents, thereby reducing the severity of seizures that is proved by pentylenetetrazole (PTZ)-induced seizures mice model (Chen L. et al., 2017; Shao et al., 2017). A study investigated and compared the pharmacokinetics of free gastrodin, parishin, and *G. elata* extract in rats. Parishin and *G. elata* extract had prolonged t_1/2_ compared with free gastrodin in rat plasma, that is 3.09 ± 0.05 h, 7.52 ± 1.28 h and 1.13 ± 0.06 h respectively, indicating that Parishin and *G. elata* extract have longer action durations than free gastrodin does (Tang et al., 2015). Matias et al. (2016) reviewed various constituents of *G. elata* related to anticonvulsant activity, including *G. elata* rhizome extracts, gastrodin, 4-Hydroxybenzyl alcohol, 4-Hydroxybenzaldehyde and analogs, vanillin, and vanillyl alcohol.

Research in 2020 revealed herb–drug interactions between *G. elata* and carbamazepine (CBZ). *G. elata* reduced the autoinduction of CBZ and increased the plasma CBZ concentration (Yip et al., 2020). These studies revealed the values of *G. elata* as an anticonvulsant drug or adjuvant therapy. However, physicians should carefully consider drug dosage and side effects, such as itching rash and poor appetite, caused by the herb–drug interaction (Yip et al., 2020).

#### Uncaria rhynchophylla

*Uncaria rhynchophylla* (UR) and *G. elata* are usually used in combination to treat convulsive disorder (Hsieh et al., 1999). They are considered an herb pair. In a kainic acid-treated rat model, UR has anticonvulsive and free radical scavenging activities and may have a synergistic effect when combined with *G. elata* that delays the onset of wet dog shakes, that is 63 min compared with 27 min in the control group, while 40 min in the *G. elata* group (Hsieh et al., 1999). Rhynchophylline is a component of UR that can treat the underexpression of macrophage migration inhibitory factor (MIF) and cyclophilin A in the frontal cortex and hippocampus in kainic acid-induced epilepsy rats. It showed UR group increased 3.1-fold MIF and 2.08-fold cyclophilin A while rhynchophylline group increased 2.75-fold MIF and 1.83-fold cyclophilin A in the frontal cortex; UR group increased 1.57-fold MIF and 1.35-fold cyclophilin A while rhynchophylline group increased 1.69-fold MIF and 1.26-fold cyclophilin A in the hippocampus, which were compared to the control group (Lo et al., 2010). Studies had reported that rhynchophylline can reduce epileptic seizures, a kainic acid-induced seizure rat model showed rhynchophylline can initiate c-Jun aminoterminal kinase phosphorylation (JNKp) in the MAPK signaling pathways (Hsu et al., 2013) as well as in a pilocarpine-induced status epilepticus rat model of temporal lobe epilepsy showed it can inhibit Na_v_1.6 persistent sodium currents (I_NaP_) and NMDA receptor currents (Shao et al., 2016). In kainic acid-induced epileptic seizures rats, UR has neuroprotective effects through reducing glial fibrillary acidic protein and S100B protein expression and inhibiting receptors for advanced glycation end products, not including GABA_A_ and transient receptor potential vanilloid subtype 1 (TRPV1) receptors. UR has also been demonstrated to attenuate mossy fiber sprouting and astrocyte proliferation and prevent hippocampal neuron death, especially in the CA1 and CA3 areas (Lin and Hsieh, 2011; Liu et al., 2012; Tang et al., 2017). Furthermore, UR regulates toll-like receptor and neurotrophin signaling pathways and inhibits the expressions of interleukin (IL)-1β and brain-derived neurotrophic factor (BDNF) genes in kainic acid-induced seizure rats’ cortex and hippocampus (Ho et al., 2014).

#### Acori tatarinowii

*Acori tatarinowii* is a type of aquatic plant that is commonly used to treat neurological, cardiovascular, respiratory, and gastrointestinal diseases. *A. tatarinowii* decoction and its volatile oil have been demonstrated that reduce seizure attacks in maximal electroshock (MES) model. The decoction of *A. tatarinowii* decreased convulsive rates in PTZ-induced seizure rats from 100% (normal saline control group) to 67% (dose 10 g/kg of decoction) while 33% in sodium valproate group. Volatile oil of *A. tatarinowii* could not decrease convulsive rates but could reduce mortality rates of pentylenetetrazol-induced seizure rats from 92% (normal saline control group) to 40% (managed with dose 1.25 g/kg of volatile oil) (Liao et al., 2005). A major ingredient of *A. tatarinowii*, α-asarone, modulates GABA_A_ receptors, enhances tonic GABAergic inhibition, and suppresses the excitability of CA1 hippocampal pyramidal neurons in PTZ and kainate mouse models (Huang et al., 2013). α-asarone and β-asarone increase the expression of neurotrophic factors, including nerve growth factor (NGF), BDNF, and glial-derived neurotrophic factor (GDNF), in cultured rat astrocytes. The expression is partially activated by triggering the cAMP-dependent protein kinase (PKA) signaling pathway (Lam et al., 2019). In the MES test and PTZ-induced seizures in mice models, eudesmin extracted from *A. tatarinowii* can increase GABA while reducing glutamate levels. Furthermore, eudesmin upregulates the expression of GABA_A_ and glutamate decarboxylase 65 (GAD65) and modulates Caspase-3 and Bcl-2, both of which are related to neuron apoptosis (Liu et al., 2015).

#### Paeonia lactiflora

*Paeonia lactiflora* can suppress the elevation of c-Fos protein and increase transthyretin and phosphoglycerate mutase 1 expression in cobalt-treated mouse cerebrum, thereby exerting a neuroprotective effect on cerebral neurons (Kajiwara et al., 2008). Paeoniflorin, the major active component of *P. lactiflora*. In a hyperthermia-induced seizure of immature rats’ model, paeniflorin suppresses the elevation of glutamate-induced intracellular Ca^2+^, which is related to metabotropic glutamate receptor 5 (mGluR5) activation. The anticonvulsive effect of paeoniflorin is not associated with the release of GABA, the regulation of a-amino-3-hydroxy-5-methyl-4-isoxazolpropionic acid (AMPA), or the regulation of NMDA receptors. It would be a possible herbal medicine for treating febrile seizure in children (Hino et al., 2012). Shosaiko-to-go-keishika-shyakuyaku-to is the Japanese Kampo medicine, and only Paeoniae radix, the main component of the formula, had significant inhibition effect of PTZ-induced EEG power spectrum changes (Sugaya et al., 1988).

#### Bupleurum chinense

*Bupleurum chinense* has various functions, including hepatoprotective, antitumor, antioxidant, antidepressant, anti-inflammatory, and anticonvulsant effects (Jiang et al., 2020). Saikosaponin a isolated from *B. chinense* showed anticonvulsive and neuroprotective effects by inhibiting NMDA receptor current, I_N__ap_, and the mammalian target of rapamycin (mTOR) signaling pathway and increasing Kv4.2-mediated A-type voltage-gated potassium currents (Kv4.2-mediated IA) that proved by rat models (Yu et al., 2012; Ye et al., 2016; Hong et al., 2018). Saikosaponin can reduce the severity and duration of seizures, and prolong the latency of seizure in PTZ-induced rats (Ye et al., 2016). Some Chinese medicine formulas containing *B. chinense*, such as “Saiko-Keishi-To (Chai-Hu-Gui-Zhi-Tang)” and a modified formula of “Chaihu-Longu-Muli-Tang,” have been reported to have anticonvulsant and antioxidant effects (Sugaya et al., 1985, 1988; Wu et al., 2002). The maintenance of calcium distribution and calcium binding state were shown in highly PTZ-sensitive snail neurons incubated in Saiko-keishi-to, and it indicated that Saiko-keishi-to has an inhibitory effect on calcium shift and binding state change (Sugaya et al., 1985). An open add-on study performed modified formula of Chaihu-Longu-Muli-Tang for 20 refractory epilepsy and 20 benign epilepsy patients 4 months, and the formula decrease seizure frequency in refractory epileptics from 13.4 ± 3.4 to 10.7 ± 2.5 per month (*p*-value was 0.084) that may be attribute to antioxidant effects with reducing serum malondialdehyde and copper-zinc superoxide dismutase (*p* < 0.05) while there are no statistically significant changes in benign epilepsy patients, that is because only refractory epilepsy group has significant variation of lipid peroxidation compared to age-matched healthy control group (Wu et al., 2002).

#### Ziziphus jujuba

*Ziziphus jujuba* is usually used to treat insomnia in traditional Chinese medicine. A study designed with the MES model and the PTZ model of rats indicated that *Z. jujuba* achieves anticonvulsant effects by increasing acetylcholinesterase (AChE) and butyrylcholinesterase (BChE) activity and the latency of myoclonic jerks, thereby preventing seizure attacks (Pahuja et al., 2011). The additional usage of hydroalcoholic extract of *Z. jujuba* can enhance the anticonvulsant effects of phenytoin and phenobarbitone but not carbamazepine that is evaluated in MES-induced seizure rats (Pahuja et al., 2012).

#### Pinellia ternata

*Pinellia ternata* is mostly used to treat ailments of the respiratory and gastrointestinal systems. A component of *P. ternate*, pinellia total alkaloids, is involved in the modulation of GABAergic systems through its increase of GABA and GAD65 expression, reduction of GABA transporter-1 (GAT-1) and GABA transaminase (GABA-T) expression, and upregulation of GABA_A_ receptor α5, δ, α4, and γ2 subunits in the hippocampal formation. Research in 2020 indicated that pinellia total alkaloids (PTA) may exert antiepileptogenic effects that reduce the occurrence of spontaneous recurrent seizures in pilocarpine-induced epileptic rats, and PTA 800 mg/kg group has the lowest frequencies of spontaneous recurrent seizures compared to PTA 400 mg/kg group and Topiramate 60 mg/kg group (Deng et al., 2020).

#### Paeonia suffruticosa

Paeonol is extracted from the root bark of peony trees and is usually used to activate blood circulation. A study in 2019 designed with five groups of PTZ-induced seizure rats, which are normal control group, epilepsy group, low-dose paeonol-treated group, medium-dose paeonol-treated group and high-dose paeonol-treated group that first explored the anticonvulsant effect of paeonol (Liu et al., 2019). Paeonol was determined to reduce the severity and duration of seizures and increase the latency of seizure. Furthermore, it protects hippocampal neurons from damage by reducing oxidative stress and inhibiting apoptosis in the CA1 areas while inhibiting the expression of the proapoptotic factor cleaved caspase-3. The seizure intensity was scored as stage 0, no response; stage 1, facial movements and ear and whisker twitching; stage 2, myoclonic convulsions without rearing; stage 3, myoclonic convulsions with rearing; stage 4, tonic-clonic convulsions; stage 5, generalized tonic-clonic seizures with loss of postural control; and stage 6, death. High-dose paeonol-treated group (60 mg/kg) reduce the seizure stage to 2.17 ± 0.41 compared to PTZ-kindled epilepsy group 4.67 ± 0.52 (Liu et al., 2019).

#### Stephania tetrandra

Tetrandrine is a voltage-gated Ca^2+^ channel blocker isolated from *S. tetrandra*. A study reported that tetrandrine regulates apoptosis and protects brain cells by increasing the expression of Bcl-2 and reducing the expression of Bax. And tetrandrine could lessen the withdrawal symptoms such as weight loss induced by phenobarbital-dependency that proved by phenobarbital-withdrawn rat model (Han et al., 2015). Other studies in multidrug resistance cells and PTZ-induced seizure rats model revealed that tetrandrine can reduce the antiepileptic drug resistance of phenytoin and valproate by reducing the expression of multidrug-resistant protein *P*-glycoprotein (*P*-gp) at the mRNA and protein levels in the cortex and hippocampus, enhancing the efficacy of antiepileptic drugs. The seizure severity assessed by standards of Racine as grade IV and V were decreased in refractory epilepsy rats which were treated with tetrandrine (Chen et al., 2015).

#### Cistanche deserticola

*Cistanche deserticola* is a type of desert plants that grows in China. Echinacoside is a compound of *C. deserticola.* Pretreated 10 or 50 mg/kg echinacoside for 30 min on kainic acid-induced seizures rats can enhance their neuronal survival and prevent epilepsy by inhibiting glutamate excitotoxicity and autophagy, suppressing inflammation, and activating protein kinase B (Akt)/glycogen synthase kinase (GSK) 3β signaling. Therefore, it significantly increased seizure latency more than 1 h and decreased seizure severity (Lu et al., 2018a). A 4-aminopyridine (4-AP)-induced epileptiform activity with an *in vitro* rat hippocampal neurons’ model study reported that echinacoside reduced spontaneous glutamate release, the frequency but not the amplitude of spontaneous excitatory postsynaptic currents, and the sustained repetitive firing of action potentials in hippocampal CA3 pyramidal neurons (Lu et al., 2018b).

#### Corydalis yanhusuo

*Corydalis yanhusuo* has analgesic effects. Furthermore, sedative, hypnotic, antihypertensive, antiepileptogenic, and anticonvulsant effects of *C. yanhusuo* have also been identified. Two studies used amygdala kindling seizures rats’ model reported that DL-tetrahydropalmatine is a component that can inhibit amygdaloid dopamine release, thus reducing epilepsy attacks (Chang and Lin, 2001; Lin et al., 2002).

#### Salvia miltiorrhiza

*Salvia miltiorrhiza* is commonly used to improve vascular circulation and has various functions, including anti-inflammatory, antioxidative, antiatherogenetic, antithrombotic, antihypertensive, antihyperlipidemic, antifibrotic, and antitumorous activities (Meim et al., 2019). Salvianolic acid B is a major water-soluble substance of *S. miltiorrhiza*. A study revealed that salvianolic acid B can reduce apoptosis and activate Akt/cAMP response element binding protein (CREB)/BDNF signaling pathways that contribute to neuronal survival and growth in the cortex and hippocampus, thus suppressing epilepsy in PTZ-kindled rats’ model. PTZ-kindled rats treated with salvianolic acid B showed lower seizure stage 3.900 ± 0.718 compared to non-treated PTZ group 4.938 ± 0.250. Salvianolic acid B significantly decreased the total seizure times across the entire experiment from 19.150 ± 5.851 to 9.600 ± 3.515, and also significantly decrease the duration of seizures from 48.200 ± 5.782s to 29.950 ± 4.442s (Yu et al., 2019). Other studies have revealed that combining compound Danshen dripping pills (CDDP) with carbamazepine (CBZ) to treat kainic acid-induced temporal lobe epilepsy reduced seizure severity and frequency through antiapoptotic effects and the upregulation of GDNF expression in the CA3 area of the hippocampus. Compared to only CPPD or CBZ treatment group, the combination of CDDP with CBZ exerted a positive interaction effect that significantly decreased seizure stage, decreased the frequency of spontaneous recurrent seizures, and preserved the most number of surviving neurons (Jia et al., 2018).

### Fungus

#### Ganoderma lucidum

In folklore, *Ganoderma* is considered a mysterious, magical, and precious Chinese medicine. A review in 2019 reported that the most commonly used Ganoderma are *G. lucidum*, *G. applanatum*, *G. sinense*, *G. tsugae*, *G. capense*, and *G. boinense* (Zhao et al., 2019). However, *G. lucidum* is the traditional and most widely known species of Ganoderma.

A study harvested and cultured primary hippocampal neurons from rats, then established the epileptiform discharge hippocampal neuron model. The study indicated that *G. lucidum* polysaccharides can inhibit the accumulation of Ca^2+^ in hippocampal neurons and stimulate Ca^2+^/calmodulin-dependent protein kinase II α (CaMK II α) expression, thus reducing neuronal excitability (Wang et al., 2014). In an epileptiform discharge hippocampal neuron model, *G. lucidum* spores inhibit the expression of *N*-cadherin, which is related to mossy fiber sprouting and synaptic reconstruction, thus suppressing the neural circuit formed by mossy fiber sprouting. *N*-cadherin also promotes neurotrophin (NT)-4 expression, which is associated with neuron survival, inhibition of apoptosis, and synaptic plasticity, and thus protects hippocampal neurons (Wang et al., 2013). Ganoderic acid is the primary component of *G. lucidum* spores. In another epileptiform discharge hippocampal neuron model, Ganoderic acid prevents the apoptosis of hippocampal neurons and enhances the expression of BDNF and transient receptor potential canonical 3 (TRPC3), which is involved in neuron plasticity and synaptic reconstruction, inhibits mossy fiber sprouting, and aids in the recovery of damaged neurons (Yang et al., 2016).

A retrospective study in 2018 included 18 patients with epilepsy who were treated with *G. lucidum* spore powder therapy three times per day for 8 weeks. The study revealed that the powder reduced the weekly seizure frequency and the severity of each seizure episode (Wang et al., 2018). Further studies are required to confirm its efficacy in treating human epilepsy.

### Animals

#### Buthus martensii

Although scorpions have various levels of toxicity, they are a staple of traditional Asian street food and medicinal wines from ancient times. Scorpions are usually used to treat neurological and musculoskeletal diseases, such as stroke, headache, seizure, and joint pain. *B. martensii* is the most abundant species of Asian scorpion and has been widely used in Chinese medicine since the Song dynasty of China. Antiepilepsy peptides (AEPs) are bioactive polypeptides extracted from its venom. AEP can easily cross the blood–brain barrier because of its low molecular weight (8.3 kDa), and it exhibits anticonvulsant effects by binding with synaptosomal-associated protein (SNAP)-25 and NMDA (Wang et al., 2009). A study demonstrated that AEP can control neuronal excitability by selectively modifying voltage-gated sodium channels in primary cortical neurons cultured from mice. AEP especially inhibits Na_v_1.6 currents in human embryonic kidney (HEK)-293 Cells, thus suppressing action potentials in neurons (Zhang et al., 2019).

#### Bombyx mori

Silkworms and their chrysalis are edible and high in protein. Infecting B. mori silkworms with the fungus *Beauveria bassiana* kills and dries the body of the silkworms. These infected silkworms are used as traditional Chinese medicine with reported anticonvulsant, anticoagulant, antitumor, antioxidant, antibacterial, antifungal, antiviral, hypoglycemic, and immunomodulatory effects (Hu et al., 2017, 2019). The anticonvulsant, hypnotic, and neurotrophic effects of some small molecule compounds, such as beauvericin and ammonium oxalate, have been explored (Hu et al., 2017). Several studies involving animal models have investigated the macromolecular compounds of *B. mori*, which had not been previously investigated. The protein-rich extracts from *B. mori* were determined to act mainly on the hippocampus CA1 region, and decreased seizure rates in MES-induced seizure mice and increased seizure and death latency in PTZ-induced seizure mice (Hu et al., 2019). The extracts protect neurons from oxidative damage and cell apoptosis by regulating the phosphoinositide 3-kinase (PI3K)/Akt signaling pathways in H_2_O_2_-stimulated PC12 cells (rat pheochromocytoma cells) *in vitro* (Hu et al., 2019). The extracts also achieve neuroprotective effects through reducing IL-1β, IL-4, and tumor necrosis factor (TNF)-α, increasing 5-HT and GABA, and reducing intracellular Ca^2+^ levels, preventing neuronal signaling, that was investigated on NGF-induced PC12 cells injured by glutamate (He et al., 2020).

#### Cryptotympana atrata

*Cryptotympana atrata*, cicada exuviae, is a commonly used traditional Chinese herb in dermatological, ophthalmological, otorhinolaryngological, and neurological diseases. *C. atrata* can be cooked as porridge and soup, or made into tea for medical diet therapy. In a study of drug (PTZ, picrotoxin, or strychnine)-induced convulsions rat model, the extracts of *C. atrata* had anticonvulsive, sedative, and hypothermic effects; water extracts were more effective than ethanol extracts (Hsieh et al., 1991).

Therefore, Chinese herbs (plant, fungi, and animals) exert anti-inflammatory, antioxidant, and neuroprotectant effects by acting on GABA, NMDA, and sodium channels, among others. The summarized possible mechanisms are presented in Table 1. These effects are helpful for treating epileptic seizures. However, randomized, double-blind controlled clinical trials to confirm the antiepileptic effects and the efficacy in the treatment of epilepsy are lacking.

### Evidence-Based Human Applications

To explore the evidence and reliability of Chinese medicine application on human, we collect and review the human clinical trials. There are four human clinical studies treating epilepsy with Chinese medicine that have been published. Three of the studies investigated the compounds of Chinese medicine, and one of the studies focused on a unique herb. Table 2 describes the details of those studies.

**TABLE 2:** Characteristics of human clinical studies.

Study	Study types	Intervention	Control	Epilepsy types	Intervention group/Control group	Age (mean ± SD or range)	Gender (M/F)	Treatment time
Wu et al., 2002	Clinical trial	Modified formula of Chaihu-longu-muli-tang (TW970) (8 mg/day)	None	Partial epilepsy	I: group A: 20 refractory epilepsy; group B: 20 benign epilepsy C: group C: 20 age-matched healthy adults	A: 25.9 ± 7.0 B: 27.5 ± 10.0	A:11/9 B: 9/11	4 m
Ma et al., 2003	Observational study	Anti-epilepsy capsules (≤5 years old: 1–5 capsules 6–10 years old: 7 capsules 11–14 years old: 8 capsules for each time, three times daily)	Luminal (1.5–2 mg/kg each time, three times daily)	Generalized and partial epilepsy	930/160	1–14	I: 518/412 C: 95/65	6 m
He et al., 2011	Randomized and controlled multicenter clinical trial	Dianxianning pills (four pills three times daily)	Placebo pills (four pills three times daily)	Generalized tonic-clonic seizures or partial seizure	141/71	I: 33.99 ± 15.05 C: 33.90 ± 16.30	I: 87/50 C: 41/28	3 m
Wang et al., 2018	Observational study	*Ganoderma lucidum* spore powder (three times daily)	None	Systemic, partial, and atypical attack of seizure	18/none	39.4 ± 15.3	10/8	8 w

*SD, standard deviation; I, intervention; C, control; m, months; w, weeks.*

Saiko-ka-ryukotsu-borei-to (Chaihu-Longu-Muli-Tang) combined with *Gastrodia elata* and *Uncaria rhynchophylla* had antioxidant effects reducing the seizure frequency in refractory epilepsy patients from 13.4 ± 3.4 to 10.7 ± 2.5 every month (Wu et al., 2002). A kind of anti-epilepsy capsule, composed of *Acorus tatarinowii*, *Arisaema cum* Bile, *Gastrodia elata*, *Pseudostellaria heterophylla*, *Poria cocos*, *Citrus reticulata*, *Pinellia ternata*, *Aquilaria sinensis*, and *Citrus aurantium*, helped to control the electric discharge of the brain and improved the signs of epileptic electric discharge shown by electroencephalography. It effectively decreased the epilepsy frequency and the duration of attack for different types of epilepsy, including infantile spasm, autonomic, complex partial, holotonic-clonic, absence, localized Rolandic, psychomotor, myoclonus and indefinite types. The total effective rate and recovery rate of the intervention group are 83.33 and 54.3%, versus 51.88 and 38.4% in the control group, respectively (Ma et al., 2003). Dianxianning Pian is produced by the Chinese medicine factory of China. The pill contains *Valeriana jatamansi*, *Acorus tatarinowii*, *Uncaria rhynchophylla*, *Pharbitis nil*, *Euphorbia lathyris*, *Valeriana officinalis*, and *Nardostachys chinensis*, and can control the frequency and severity of refractory epilepsy as an adjunctive therapy. The average seizure rate decreased 37.84% in the intervention group while but 13.18% in the control group, and the epilepsy frequency gradually reduced with increased treatment time (He et al., 2011).

A study in 2018 explored the efficacy of *Ganoderma Lucidum* spore powder for treating epilepsy patients. The herb powder could reduce average weekly seizure frequency from 3.1 ± 0.8 to 2.4 ± 1.2, but it didn’t show significant differences of duration of epilepsy and quality of life. The most common adverse effect is nausea, the second one is stomach discomfort, then the others are vomiting, dizziness, dry mouth, diarrhea, sore throat, and epistaxis in order (Wang et al., 2018).

## Other Natural Medicine

A review article in 2018 reported eighteen anticonvulsant herbal agents, including Uncaria rhynchophylla, Gastrodia elata, Cannabis, Desmodium triflorum, Viscum album, Morus alba, Berberis integerrima, Mussaenda philippica, Justicia pectoralis, Gladiolus dalenii, Ficus religiosa, Withania somnifer, Lobelia nicotianaefolia, Marsilea quadrifolia, Passiflora incarnata, Mondia whitei, and Phytol (Manchishi, 2018). He et al. (2021) reviewed the bioactive compounds of natural drugs and categorized their antiepileptic mechanisms. There were 38 compounds could regulate neurotransmitters and synaptic function, 16 compounds regulate ion channels and the ion flow, 15 compounds modulate the immune system and exert anti-inflammatory effect, 5 compounds inhibit the activation of glial cells, 19 compounds exert anti-apoptotic and anti-oxidant effect by protecting neurons from mitochondrial damage and oxidative stress, and 25 compounds treat epilepsy by other mechanisms (He et al., 2021). The review article mentioned natural drugs such as Salvia miltiorrhiza Bunge, Curcuma longa, Passiflora caerulea, Matricaria chamomilla, Scutellaria baicalensis, Bupleurum chinense, Plantago asiatica, Camellia sinensis, Withania somnifera, Maclura tinctoria, Radix astragali, Citrus reticulata Blanco, Cotinus coggygria, Gastrodia elata Blume, Radix bupleuri, Lantana camara, Curcuma longa, Rhododendron tomentosum, Matricaria chamomilla, Dennettia tripetala, Nigella sativa, Thymus vulgaris, Ginkgo biloba, Capsicum annuum, Crocus sativus, Uncaria rhynchophylla, Piper nigrum, Acorus tatarinowii, and so on. Among the natural medicine, cannabidiol inhibits neuronal excitability and exerts anticonvulsant properties, and it has been the first product made directly from the cannabis plant that approved by the United States Food and Drug Administration in 2018 (Lattanzi et al., 2018).

## Herb–Drug Interaction

The combination therapy of anti-epileptic drugs and herbs are more and more popular and acceptable nowadays. One of the difficulties to confirm the herb–drug interaction is due to the complex ingredients of one herb, or there are many herbs in a formula of Chinese medicine. Some natural herbs interact with anti-epileptic drugs then enhance the anti-convulsive effects were reported. There are few studies investigated the herb-drug interactions and the possible mechanisms (Pearl et al., 2011; He et al., 2021). In a PTZ-induced seizure mice model, Nobiletin and Clonazepam reduce seizure severity by regulating the balance of glutamate and GABA, modulating GABA_A_ and GAD 65, inhibiting apoptosis, inhibiting BDNF-TrkB signaling pathway and activating PI3K/Akt signaling pathway (Yang et al., 2018). The combination of Naringin and Phenytoin in PTZ-kindled rats could significantly decrease the seizure scores, elevate GABA and dopamine, decrease glutamate, against oxidation, and protect neurons in PTZ-induced seizure rats (Phani et al., 2018). Umbelliferone combined with phenobarbital or valproate elevate the threshold of electroconvulsions and enhance the anti-convulsive efficacy in MES-induced seizure mice model (Zagaja et al., 2015). In an oral CBZ rat model, sinapic acid inhibits hepatic cytochrome P450 3A2, 2C11, and intestinal *P*-glycoprotein then increases the absorption of CBZ (Raish et al., 2019).

Those studies reported natural medicines, most of them are plants and herbs, can improve the efficacy of anti-epileptic drugs. In some studies, Chinese medicine showed the positive effect in combined therapy with Western medicine that have been mentioned in this review article. There are rare studies explore the adverse effects of the combined therapy. It still needs more well-designed studies to investigate the herb–drug interactions of Chinese medicine combined with anti-epileptic drugs because of the insufficient evidences. Patients who use herbs as adjuvant therapy should inform their doctors to prevent the side-effects or complications that may cause by the potential herb–drug interactions.

## Limitations

We reviewed Chinese medicine that is mainly used in clinical treatment in epilepsy, but other potential herbs may not be reviewed due to less references investigated. Some natural drugs include Chinese medicine are lacking of large amounts of evidences to confirm the anti-epileptic effects. In addition, most studies are explored the efficacies and mechanisms of Chinese medicine in treating epilepsy but less mentioned its side effects. It still also lack researchers to devote their efforts to the herb-drug interaction and side effects of Chinese medicine. As searching the databases, we found there are large portions of cell and animal models in studying the anti-epileptic effect of Chinese medicine, but the human clinical trials are extremely deficient. For the safety and efficacy of Chinese medicine and natural drugs in evidence-based practice, the further well-designed randomized controlled trials are promptly needed.

## Conclusion

Anticonvulsive herbs used in clinical settings to treat epilepsy and seizure are discussed in the present article and their possible antiepileptic mechanisms, including anti-inflammation, antioxidation, GABAergic effect enhancement, NMDA receptor and sodium channel modulation, and neuroprotection, were described. Although some anticonvulsive effects of herbs have been reported, there are remaining the issues of the appropriate treatment time, dosage of herbs, and the long-term effects after the intervention. Larger sample size, high-quality randomized controlled clinical trials and adequate experimental evidences to confirm their antiepileptic effects are lacking. Therefore, further study is warranted.

Chinese medicine is holistic and can be personalized for individual patients based on their symptoms. Medical diet therapy using traditional Chinese medicine has spread globally. Herbal medicine is used as adjunctive therapy or main therapy in some countries, especially in East. The future directions of the use of herbal medicine are evidence-based practice that needs further high-quality clinical trials for proving its efficacy and safety, and the concerns of herb-drug interaction as combined therapy with Western medicine. Some Chinese herbs have been proved the anti-epilepsy effects and have the potential to improve the access to effective treatment and to avoid the adverse effects of Western medicine for epilepsy patients. The combined use of different herbs exerts an anticonvulsant effect through various mechanisms. But for the safety of combined therapy with Western medicine, patients should consult and inform their physicians to notice the probable side effects caused by herb–drug interactions.

## Author Contributions

C-HL and C-LH: conceptualization. C-HL: data curation and writing—original draft preparation. C-LH: writing—review and editing. Both authors read and agreed to the published version of the manuscript.

## Conflict of Interest

The authors declare that the research was conducted in the absence of any commercial or financial relationships that could be construed as a potential conflict of interest.
